# Challenges and strategies for *in situ* endothelialization and long-term lumen patency of vascular grafts

**DOI:** 10.1016/j.bioactmat.2020.11.028

**Published:** 2020-12-05

**Authors:** Yu Zhuang, Chenglong Zhang, Mengjia Cheng, Jinyang Huang, Qingcheng Liu, Guangyin Yuan, Kaili Lin, Hongbo Yu

**Affiliations:** a Department of Oral and Cranio-maxillofacial Surgery, Shanghai Ninth People's Hospital, College of Stomatology, Shanghai Jiao Tong University School of Medicine, Shanghai, 200011, China; b National Clinical Research Center for Oral Diseases, Shanghai, 200011, China; c Shanghai Key Laboratory of Stomatology & Shanghai Research Institute of Stomatology, Shanghai, 200011, China; d National Engineering Research Center of Light Alloy Net Forming & State Key Laboratory of Metal Matrix Composite, Shanghai Jiao Tong University, 800 Dongchuan Road, Shanghai, 200240, China

**Keywords:** Vascular graft, *In situ* endothelialization, Thrombogenesis, Intimal hyperplasia, Immunomodulation

## Abstract

Vascular diseases are the most prevalent cause of ischemic necrosis of tissue and organ, which even result in dysfunction and death. Vascular regeneration or artificial vascular graft, as the conventional treatment modality, has received keen attentions. However, small-diameter (diameter < 4 mm) vascular grafts have a high risk of thrombosis and intimal hyperplasia (IH), which makes long-term lumen patency challengeable. Endothelial cells (ECs) form the inner endothelium layer, and are crucial for anti-coagulation and thrombogenesis. Thus, promoting *in situ* endothelialization in vascular graft remodeling takes top priority, which requires recruitment of endothelia progenitor cells (EPCs), migration, adhesion, proliferation and activation of EPCs and ECs. Chemotaxis aimed at ligands on EPC surface can be utilized for EPC homing, while nanofibrous structure, biocompatible surface and cell-capturing molecules on graft surface can be applied for cell adhesion. Moreover, cell orientation can be regulated by topography of scaffold, and cell bioactivity can be modulated by growth factors and therapeutic genes. Additionally, surface modification can also reduce thrombogenesis, and some drug release can inhibit IH. Considering the influence of macrophages on ECs and smooth muscle cells (SMCs), scaffolds loaded with drugs that can promote M2 polarization are alternative strategies. In conclusion, the advanced strategies for enhanced long-term lumen patency of vascular grafts are summarized in this review. Strategies for recruitment of EPCs, adhesion, proliferation and activation of EPCs and ECs, anti-thrombogenesis, anti-IH, and immunomodulation are discussed. Ideal vascular grafts with appropriate surface modification, loading and fabrication strategies are required in further studies.

## Highlights

• This paper reviews challenges and strategies for enhanced *in situ* vascularization.
• Strategies for homing, adhesion and activation of EPCs and ECs are summarized.
• Approaches to prevent thrombogenesis and IH for long-term lumen patency are introduced.
• Immune system plays a role in modulating biological behaviors of ECs and SMCs.
• Perspectives for future development of vascular grafts are proposed.

## Graphical abstract

**Figure:**
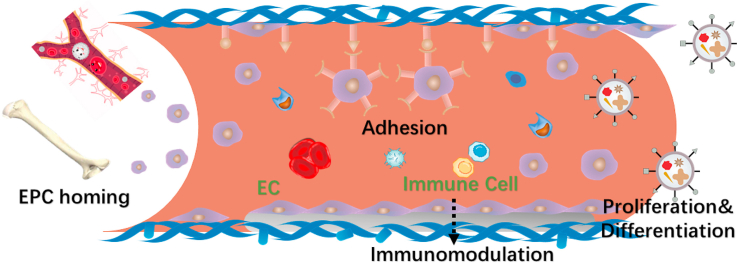

## 1 Introduction

Vascular diseases are the most prevalent cause of ischemic necrosis of tissue and organ, which has attracted much attention [1]. Vascular defect caused by trauma or underlying diseases like diabetes can reduce oxygen and nutrients supply for tissues and organs, which may result in severe consequences, like claudication, sores, organ disfunctions, necrosis, or even death [2,3]. When long-segment defects occurred or the defects happened in vital organs like heart, artificial vascular grafts are required to restore blood supply for tissues.

Synthetic vascular grafts have been widely utilized in clinics as conventional strategies for vascular impairment, like polyurethane, polyester, expanded polytetrafluoroethylene (ePFTE), and etc., with diameter greater than 6 mm [4]. However, these synthetic grafts have long-term risk since they are prone to intimal hyperplasia (IH) and thrombogenesis, and result in implantation failure [5], particularly for small diameter vascular grafts (diameters less than 4 mm) [6]. Hence, ideal vascular grafts are required to imitate the framework and constitution of native vessels, as well as inhibit protein deposition, blood coagulation, and immunological rejection [7,8]. To construct a biomimetic vascular graft, it is indispensable to figure out the critical factors and challenges in graft development.

It has been widely recognized that endothelialization is critical for blood contacting devices [9,10]. The endothelium, the inner tunica with monolayer endothelial cells (ECs) lining in vessel lumen, directly contacts with blood, and plays an important role in maintaining vascular hemostasis and patency by releasing regulatory molecules including nitric oxide (NO), heparins, and plasmin, etc. [9]. Losing endothelium layer may lead to a cascade of pathological reactions, like thrombogenesis, inflammation reactions, and smooth muscle cell (SMC) hyperplasia [11,12]. Thus, endothelium regeneration is crucial for vascular graft.

In conventional tissue engineered vascular grafts (TEVGs), ECs are cultured and seeded on scaffolds prior to implantation, which is called *in vitro* endothelialization [13]. The proliferation ability of *in vitro* cultured ECs is limited. And greater stemness stem cells are applied, like endothelial progenitor cell (EPC), induced Pluripotent Stem Cell (iPSC), and mesenchymal stem cell (MSC) [[14], [15], [16], [17]]. However, the viability, bioactivity and stability of seeded cells after implantation cannot guarantee, and the clinical application of this strategy is inhibited by its poor effectiveness and practicality [18,19]. Moreover, *in vitro* cell culture consumes more time and cost, and have greater risk of contamination. *In situ* endothelialization, commanding the regeneration of a healthy endothelium on the surface of vascular grafts directly after implantation, is more effective than *in vitro* endothelialization [20,21]. Early strategies pay attention to appealing cells from anastomotic regions, but poor EC proliferative ability hinders the long-term expectation. Thus, the mobilization, recruitment and homing of EPC from peripheral blood and bone marrow has appealed much attentions [22,23]. Furthermore, ideal *in situ* endothelialization needs more attention on biomaterial type, surface modification and releasing factors to regulate cell performance, in which enhanced adhesion, orientation, proliferation and activation of ECs and EPCs on graft surface are required.

For long-term patency, thrombogenesis is the key factor leading to vascular occlusion. Vascular grafts, as the foreigner directly exposed to blood flux in vasculature, are easy to cause protein deposition and provoke thrombogenesis [24,25]. The aggregation of insoluble fibrin, platelet, and red cells induces the coagulation cascades and results in thrombus formation [[26], [27], [28]]. ECs serve as the first line for thrombogenesis. ECs can release and control key molecules, including tissue plasminogen activator (tPA), anti-thrombins, and plasmin, etc., to modulate anti-thrombogenesis process for vascular grafts, and these molecules are also potential treating drugs for application [24,29]. Another high potential risk is IH. The platelets, inflammatory cells, and SMCs aggregate and release growth factors, resulting in SMCs proliferating and migrating to vascular intima uncontrolledly, which exerts adverse effects on lumen patency [30].

Furthermore, inflammatory responses, induced by vascular graft implantation, are crucial in modulating graft development [31,32]. Biomaterial degradations act as stimulus, activate toll-like receptors (TLRs), and thus induce initial inflammation responses, which then cause white blood cells including neutrophils, monocytes, and lymphocytes to infiltrate from blood flow to implantation scaffolds [[33], [34], [35]]. And then growth factors released by macrophages, like tumor necrosis factor-α (TNF-α), TNF-β, and interleukin-1 (IL-1), etc., may play a role in influencing the biological behavior of ECs and SMCs, and thus modulating *in situ* endothelialization and lumen patency of vascular graft [36,37].

**Fig. 1:**
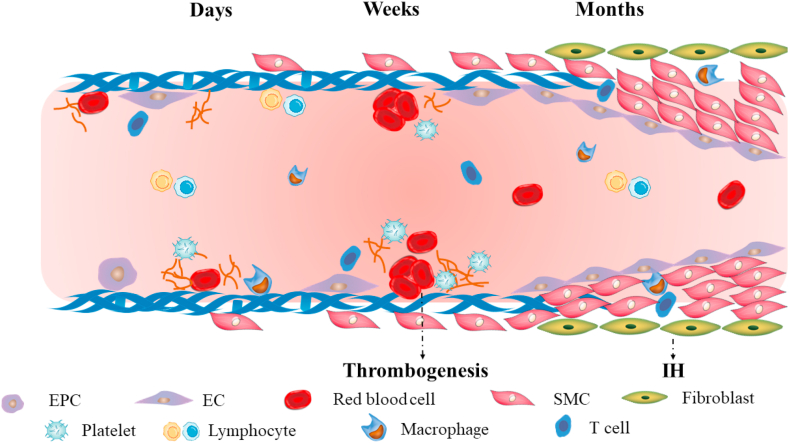
The challenges after vascular graft implantation. Days and weeks after implantation, insufficient endothelialization and thrombogenesis may happen. Months after implantation, uncontrollable proliferation of SMCs may lead to IH. Inflammatory cells play a role in regulating EC and SMC behavior.

Maintaining long-term lumen patency for small-diameter vascular graft is still challenging. Days after graft implantation, the proliferation of ECs happens, and simultaneously blood cells, platelets and fibrin deposit onto foreign graft. Weeks after implantation, more ECs proliferate and adhere onto graft surface, but no intact EC layer forms, and coagulation cascades may be activated, then resulting in thrombogenesis. Months after implantation, SMCs migrate from anastomotic sites and proliferate uncontrollably, thus leading to IH. Meanwhile, inflammatory cells, especially macrophages, can regulate EC and SMC behavior via inflammatory factors. The process is shown in Fig. 1.

**Fig. 2:**
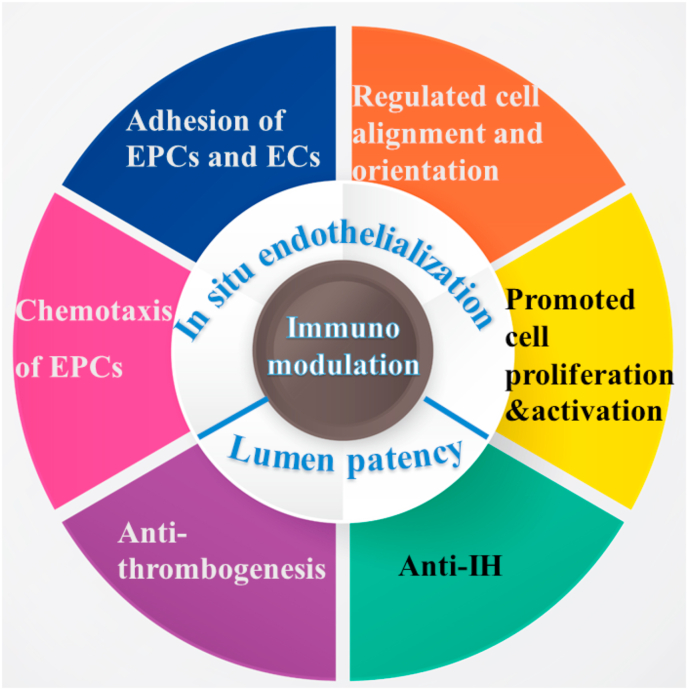
Schematic illustration for *in situ* endothelialization and lumen patency strategies.

Multiple strategies can be adopted to respond to the challenges described above for enhanced long-term lumen patency of vascular grafts (Fig. 2). ECs form the inner endothelium layer, and are crucial in preventing coagulation and thrombogenesis. Thus, promoting *in situ* endothelialization in vascular graft remodeling takes top priority, which requires recruitment of EPCs, migration, adhesion, proliferation and activation of EPCs and ECs. Surface modification with heparin or hydrophilic polymers can reduce thrombogenesis, and some drug release can inhibit IH. Additionally, NO and macrophages also play a crucial role in regulating the biological behavior of ECs and SMCs. Thus, in this paper we will review and summarize different strategies in promoting *in situ* endothelialization and inhibiting thrombogenesis and IH for long-term lumen patency of vascular grafts.

## 2 Homing and adhesion of EPCs and ECs for enhanced *in situ* endothelialization

**Fig. 3:**
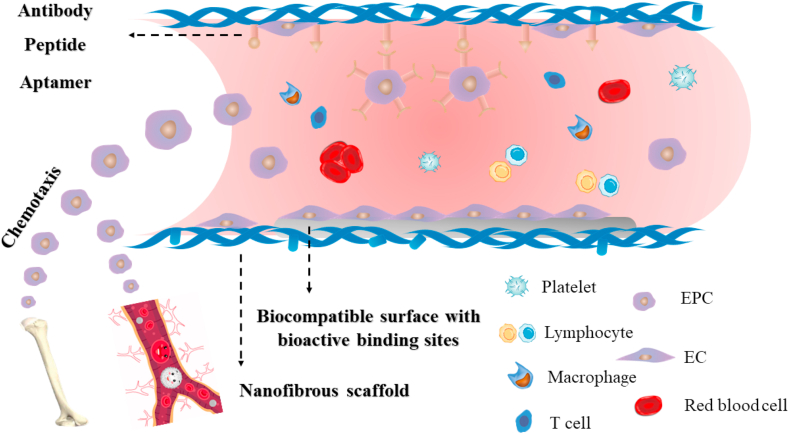
Recruitment and adhesion of EPCs and ECs. Chemokines can be utilized for EPC chemotaxis. Nanofibrous structure, biocompatible surface with bioactive binding sites and specific molecules modification can be applied for EPC and EC adhesion.

EPCs, circulating cells located in bone marrow and low quantities in peripheral blood, can differentiate into ECs [22,38]. EPCs has also been applied in cell therapy for the treatment of critical limb ischemia [39]. It has been recognized that EPCs plays a critical role in endothelialization of vascular grafts [40]. However, the quantity of EPC homing to the neovascularization sites is limited. For enhanced *in situ* endothelialization, the homing of EPCs and recruitment of ECs is vital, which includes chemotactic effects, capture and adhesion of cells on graft surface [41]. Multiple chemokines can be utilized for EPC chemotaxis, and strategies like nanofibrous structure, biocompatible surface with bioactive binding sites and specific molecules modification can be applied for cell adhesion (Fig. 3).

### 2.1 Homing of EPCs by chemokines

EPCs have multiple sub-populations and display different markers on their surface. Early EPCs display markers including CD34, CD133 and VEGFR2, etc., which are reduced as cell maturity increases [39,42]. These EPCs in different subpopulations exert synergistic effects on endothelialization [43,44]. Multiple chemokines that can be utilized for EPC homing are summarized in Table 1.

**Table 1:** Chemokines for stem cell chemotaxis to enhance *in situ* endothelialization.

Targeting Receptors		Chemokines	Loading approach	Targeting cells	Activated Signaling Pathway		Effects	Ref
CXC Family	CXCR4	SDF-1α	/	CD117^+^ stem cell	SDF-1α/CXCR4 axis		CD117^+^ cell homing to injured sites	[47,48]
	CXCR4	SDF-1α	Coating on synthetic polyester grafts	CD117^+^/CD34^+^ stem cell	SDF-1α/CXCR4 axis		CXCR4^+^ cell homing for *in situ* endothelialization	[50]
	CXCR7	SDF-1α	Immobilized onto heparin	CD34^+^ EPC, SMPC	SDF-1α/CXCR7 axis		Both EPC and SMPC recruitment for *in situ* endothelialization	[51]
	CXCR7	Dkk3	Co-electrospinning technology	Sca-1^+^ cells	Dkk3/CXCR7/ERK1/2; PI3K/AKT axis		EPC recruitment and differentiation	[52]
Integrin family	α4-integrin-VCAM1	Fibronectin (Fn)	Coating on synthetic polyester grafts	CD117^+^/CD34^+^ stem cell	Fn/VCAM1 axis		VCAM^+^ cell homing	[50]
	Integrin	8-pCPT-2′-O-Me-cAMP	/	EPC	GTPase Rap1		EPC recruitment	[59]
Others	RAGE	HMGB1	/	EPC	integrin-dependent adhesion of EPCs		EPC homing to injured sites	[60]
	Rac1	MCP1	/	SMC		p115 RhoGEF/Rac1 GTPase pathway	SMC migration and proliferation for vascular remodeling	[61]
	VEGFR1, VEGFR2	VEGF	Surface‐fixed on styrenated gelatin gel	EPC	VEGF/VEGFR		Graft for *in situ* EPC homing and capture	

The quantity of EPCs in circulation blood flow is low, and EPCs from bone marrow can mobilize into peripheral blood for enhanced endothelialization. Multiple growth factors play a role in the chemotaxis of EPC homing to the region of neovascularization, but the underlying mechanism of signaling pathways in EPC homing has not been elaborated.

#### 2.1.1 Chemokines targeting CXC families on EPC surface

Stromal cell-derived factor-1α (SDF-1α), which can act as the chemoattract for CXC family, has potential in EPCs chemotaxis and recruitment [[45], [46], [47]]. SDF-1α can bind to CXCR4 on hematopoietic stem cell (HSC) surface for stem cell homing, and it has also been reported that SDF-1α can bind to CXCR4 expressed on EPC surface [[48], [49], [50]]. Yu et al. [51] immobilized SDF-1α on vascular graft and found that SDF-1α immobilization could recruit EPCs and smooth muscle progenitor cells (SMPCs) simultaneously for enhanced *in situ* endothelialization. The *in vivo* results indicated that the lumen patency 12 weeks after implantation for naked graft was 44%, heparin coated graft was 67%, and SDF-1α/heparin modified graft was 89%. Issa et al. [52] studied the performance of dickkopf-3 (Dkk3) and results indicated that Dkk3 could interact with cell surface ligand CXCR7, activate ERK1/2 and PI3K/AKT signaling pathway, and thus enhance the recruitment and differentiation of EPCs.

Chemokines can directly immobilize on graft surface, for example, Wang et al. [53] immobilized SDF-1α onto PLLA/PLGA/PLCL vascular scaﬀolds for EPC homing. Nanoparticles (NPs) are also ideal strategy for bioactive molecules carrier. He et al. [54] constructed chitosan/fucoidan NPs to load SDF-1α to induce EC migration for promoted *in situ* endothelialization.

#### 2.1.2 Chemokines targeting integrin families on EPC surface

It has been reported that the integrins play a role in homing of EPCs. De Visscher et al. [50] constructed a synthetic graft coated with fibronectin (FN) and SDF-1α, and proved that FN could activate α4-integrin-VCAM1/FN axis for EPC homing. Particularly, integrinβ2 have potential in regulating EPC recruitment to ischemic sites [55,56]. Chavakis et al. [55] found that integrinβ2 not only induced the adherence of EPCs to ECs and ECM proteins, but also regulated the chemotaxis of EPCs to the neovascularization sites. Furthermore, integrinβ2 activated by specific anti-β2-integrin antibody can effectively enhance the homing of EPCs for *in situ* endothelialization. Integrin mediated migration of EPCs can be promoted by enhancing the activity of GTPase Rap1, and Rap1 can be activated by Epac1 [57,58]. Carmona et al. [59] utilized 8-pCPT-2′-O-Me-cAMP to directly activate Rap1 for recruitment of EPCs, providing a new strategy for promoted homing of EPCs. .

Moreover, some other molecules have also been explored and found to be able to promote homing of EPCs, like ephrine-B2-Fc chimera [62] and HMGB-1 [60], which target to specific receptors on EPC surface.

The markers like CD34 and CD133 on EPC surface are not specific, which may also display on hematopoietic stem cell (HSC) surface. Lacking specific makers on EPC surface for chemokines reduces homing efficiency. Moreover, the mechanism under chemokines induced stem cell homing has not been clearly figured out. Deep explorations are needed to uncover the mechanism, and more specific chemokines are required to effectively promote homing and recruitment of EPCs for endothelialization.

### 2.2 Adhesion of EPCs and ECs on graft surface

After homing of EPCs, cell adhesion on graft surface utilizing nanofibrous structure, biocompatible surface with bioactive binding sites or specific molecules modification takes prior consideration (Fig. 3).

#### 2.2.1 Biomimetic nanofibrous scaffolds for enhanced cell adhesion

Extracellular matrix (ECM), with nanoscale construction, is the micro-environment for cell adhesion, proliferation and differentiation, and is essential for the maintenance of cell biological activity [[63], [64], [65]]. Biomimetic nanofibrous scaffolds, with greater surface to volume ratio, provide more binding ligands for cell adhesion and biomolecules adsorption [66,67]. Multiple approaches can be applied to obtain nanoscale vascular grafts. Generally, there are three approaches for nanofiber fabrication in vascular graft construction, including self-assembly, phase separation, and electrospinning, etc.

Self-assembly is a fabrication process by which individual components are arranged into hierarchical organized structures spontaneously supported by non-covalent interactions [68]. This process is ubiquitous in various molecular bio-behavior [3,69]. Peptide amphiphiles (PAs) have been widely applied in fabricating collagen-like adaptable nanofibrous biomaterials utilizing self-assembly strategy [70,71]. Nanofibrous structure developed by self-assembly can simulate the ECM with the lowest scale at 5–8 nm, but the synthesis process is difficult to be regulated and the efficiency of productivity is relatively low. Thus, the application of this strategy in vascular graft construction is limited [72]. Phase separation can also fabricate nanofibers at the size similar to native ECM collagens, with different porous structures at macroscale [73], which can also effectively enhance cellular adhesion onto the scaffold surfaces [74]. Various biodegradable aliphatic polyesters can be developed into nanofibers using phase separation, with fiber diameter ranging between 50 and 500 nm [72,75,76]. The porosity and pore sizes can be tuned by modulating parameters, like polymer concentrations, porogen morphology utilized, gel temperatures and frozen temperatures [73]. Compared to self-assembly strategy, phase separation is a relatively simpler strategy that does not need professional techniques. However, its application is restricted because of low yield efficiency, a finite number of selected polymers for fabrication and consequently inappropriate for industrialized scale manufacture [75].

Electrospinning has been considered as an appealing strategy to simulate native ECM, for its simplicity and scalability. To fabricate optimal electrospun scaffolds, construction parameters including voltage, solution concentration, interval to collector, and collection approaches, can be tailored to acquire ideal fibrous orientation, diameters, porosity and mechanic characteristics [[77], [78], [79], [80]]. Compared to self-assembly and phase separation, a broader spectrum of biomaterials can be produced into nanofibers using electrospinning. Furthermore, convenient preparation methods, abundant biomaterials with electrospin-ability and high yield efficiency make the electrospinning strategy accessible for scaffold fabrication both on laboratory and industrial scales [75,81].

Multiple biomaterials have been utilized in electrospinning, including synthetic polymers, natural polymers and hybrid biomaterials. Non-degradable synthetic polymers like poly (ethylene terephthalate) (PET) [82,83], expanded poly (tetrafluoroethene) (ePTFE) [[84], [85], [86], [87]], polyurethane (PU) [5,88,89] have ideal electrospinnability and outstanding mechanical characteristics. Non-degradable biomaterials can be implanted for long term application, but excellent tissue engineered grafts should possess proper biodegradability for minimized inflammatory reactions. Degradable biomaterials are favorable for adhesion and proliferation of ECs [90,91]. Biodegradable synthetic polymers like poly (ε-caprolactone) (PCL) [92,93], PGA [94], PLGA [95], and natural polymers like collagen [96,97], elastin [98], silk fibroin [99], gelatin [100], are also applied for vascular graft fabrication. Natural polymers have ideal biocompatibility, but insufficient mechanical properties. It is an appealing approach to blend the natural polymers possessing outstanding biocompatibility with the synthetic polymers possessing adequate mechanical properties to overcome these disadvantages, for example, collagen and PEG [96], elastin and PCL [98], gelatin and PVA [100], etc. For construction of biomimetic vessel structure, layer by layer (LBL) strategy can be applied in vascular graft fabrication, for example, silk fibroin as inner layer, hydrogels as the medial, and TPU nanofibers as outer layer, to simultaneously obtain mechanical strength and biological activity, and simulate natural vessel structure [101].

#### 2.2.2 Biocompatible surface with bioactive binding sites for enhanced cell adhesion

Synthetic polymers can provide sufficient mechanical strength in vascular graft construction, but inadequate bioactivity since lacking sufficient cellular recognition sites for cell adhesion [102]. Natural polymers with outstanding biocompatibility can provide enough cellular ligands [103]. Thus, surface modification with natural polymer for synthetic polymers is an effective strategy for enhanced cell adhesion in vascular graft fabrication.

Gelatin possesses cell surface binding ligands RGD which are favorable for cell adhesion. Gelatin has attracted much attention for surface modification because of its biocompatibility, biodegradability, and editability. Cationized gelatin can be covalently grafted on electrospun PLLA nanofibers for better surface bioactivity [104]. Merkle et al. [105] applied co-axial electrospinning to construct core-shell structure, with PVA as core to provide mechanical strength, and with gelatin as shell to display biocompatible surface. The Young's modulus of core-shell structure was about 169 MPa and the tensile strength was about 5.4 MPa, and the mechanical properties was greatly enhanced compared with single PVA or gelatin [105]. Blended gelatin and heparin can also be an alternative strategy. Wang et al. [106] fabricated gelatin, heparin NPs, and polylysine nano-coating using self-assembly to construct a biomimetic vascular structure.

Collagen, component of natural ECM, possesses excellent biocompatibility and bioactivity [107]. Grus et al. [108] fabricated polyester vascular scaffold coated with collagen to enhance biocompatibility and improve lumen long-term patency. Furthermore, vascular graft PolyMaille (Perouse Medical, France) with collagen coating is already available. Some other natural polymers, like elastin [109], silk fibrin [110,111], are also alternative for vascular graft application. These polymeric coatings can provide non-specific binding sites not only for EC adhesion, but also for other blood cells like platelets, white cells, and SMCs, which may induce thrombogenesis and IH. Thus, more specific binding coatings are required for *in situ* endothelialization.

#### 2.2.3 Cell-capturing molecules on surface for enhanced cell adhesion

EPCs can differentiate into ECs and generate the inner endothelium layer on graft surface. Hence, it is important to targetedly capture the circulating EPCs and ECs onto graft surfaces for enhanced *in situ* endothelialization. To promote the migration and adhesion of cells, antibodies, cell adhesive peptides and cell-specific aptamers have been extensively investigated [23,112,113].

CD34 and VEGFR-2 are the surface markers on EPC surface [39]. Anti CD34 antibodies (Ab) have been the most widely utilized for EPC target and capture [114,115]. But there are some disadvantages concerning about anti CD34 Abs, since anti CD34 Abs are not only specific for EPCs, they can also capture other cells, some of which can even differentiate into SMCs and lead to thrombosis [116,117]. Clinical investigations indicated that anti CD34 Abs coated graft cannot reduce risk of vascular occlusions than conventional graft surface, especially of IH [118,119]. To overcome this problem, it was proposed that anti CD34 Abs can combine with drugs like sirolimus to reduce IH [120,121]. Anti VEGFR-2 Abs are also alternative for EPC capture. Anti VEGFR-2 Abs can effectively target and capture EPCs and ECs from blood flowing [122,123]. It has been reported that the specificity of VEGFR is superior than that of CD34 and CD31. Although the superior specificity, the VEGFR-2 also expresses on surface of monocytes and macrophages, and the recruitment of immune cells will induce undesirable inflammatory responses. The specific markers for stem cell recognition remains unclear and needs more explorations.

Cell adhesive peptides are also critical for biological identification between cell membrane and relevant ligand for cell capture and adherence. Integrins on cell surface mediate the adhesion of cell onto ECM in a dominated manner [124]. Multiple peptide sequences have been applied for surface modification for enhanced adhesion of EPCs and ECs, including RGD [[125], [126], [127], [128], [129]], CAG [130,131], REDV [132,133], and YIGSR [134,135], etc. These peptide sequences modified on graft surface display specific affinity with ECs, enhancing the adhesion of ECs and inhibiting the adherence of platelets [[136], [137], [138], [139]].

Aptamer, the short oligonucleotide sequences, exhibits affinity to specific targeted molecules. Aptamer sequences can be obtained through cell-SELEX technology [140]. It was reported that aptamer could capture porcine EPCs for enhanced *in situ* endothelialization [141]. But the application of aptamers in EPC capture has not been widely used. The effects of aptamers on the *in vitro* and *in vivo* cell performance and vascular patency still require more studies to verify. The stability of aptamers *in vivo* is unclear. Moreover, some aptamers have risks of causing inflammation responses, and the relative immunomodulation is poor known [142].

The cell-adhesive peptides, originating from ECM, bind to integrins on EC surface for cell capture. The ECs possess stronger specificity to peptides like YIGSR and REDV than SMCs. Peptides can promote cell adhesion, but with poor targeting. Antibodies can target to relevant markers on cell surface, but the targeting markers are not specific on EPC or EC surface. Aptamers, the oligonucleotide sequence, have high affinity with target cells. However, the immune responses of aptamers *in vivo* remain unclear.

## 3 Cell behavior regulation for enhanced *in situ* endothelialization

Ideal vascular graft is also required for enhanced cell elongation, proliferation, activation and differentiation of EPCs and ECs.

Circulating EPCs can be subdivided into two main categories, hematopoietic lineage EPCs and nonhematopoietic lineage EPCs. The hematopoietic EPCs originate from bone marrow and represent a provasculogenic subpopulation of hematopoietic stem cells (HSCs), which play an indispensable role in vascular repair. Different stem cells have different stemness. Embryonic stem cells (ESCs) possess totipotent differentiation potential, and mesenchymal stem cells (MSCs) are also pleuripotent in osteogenic, chondrogenic and adipogenic differentiation. But the stemness and differentiation potential of EPCs, the somatic stem cell, is limited, without multiple differentiation potential. Under normal physiological conditions in adults, the stem cells are in quiescent conditions, and maintain a dynamic balance in growth and decay of tissues. But under pathological conditions or external inductions, the ability to differentiate, regenerate and re-new can be activated. Thus, after the circulating EPCs are recruited, the defected vascular environment will release the molecules, and initiate the oriented differentiation of EPCs into ECs. To further enhance the proliferation and oriented differentiation of EPC to EC for promoted endothelialization, topography to regulate cell orientation, bioactive molecules and therapeutic genes can be applied.

### 3.1 Regulated cell alignment and orientation

Topography cues for vascular graft surface can exert significant effects on modulating biological behaviors of ECs and endothelialization [143,144]. Micro- and nano-scale topography factors including aligned nanofibers and surface patterns can induce the formation of uniformly aligned ECs for intima construction [145,146].

#### 3.1.1 Aligned nanofibers

Electrospinning technology has been widely applied in nanofibrous vascular graft fabrication, and fiber orientation can be tuned by regulating spinning parameters. The aligned nanofibers can induce cell orientation and modulate cell morphologies and biological performances [93]. Multiple studies have reported that the axially oriented fibers can arrange the morphology and alignment of ECs or MSCs for intima reconstruction [147,148]. Furthermore, the aligned fibers can also provide tensile mechanical strength, enhance SMC alignment in outer layer [149], and higher lumen patency rate [150].

#### 3.1.2 Surface micro- and nano-patterns

Cell-surface interactions play a crucial role in enhancing endothelialization. Graft surface with micro-/nano-scale grove/ridge patterns can enhance the construction of an intact EC layer spontaneously aligned along a prior orientation, which then effectively simulates the elongated endothelium layer structure [151,152]. The aligned ECs have been reported to inhibit leukocyte invasion and consistent with elongated morphology of native ECs under blood flow [153,154].

**Fig. 4:**
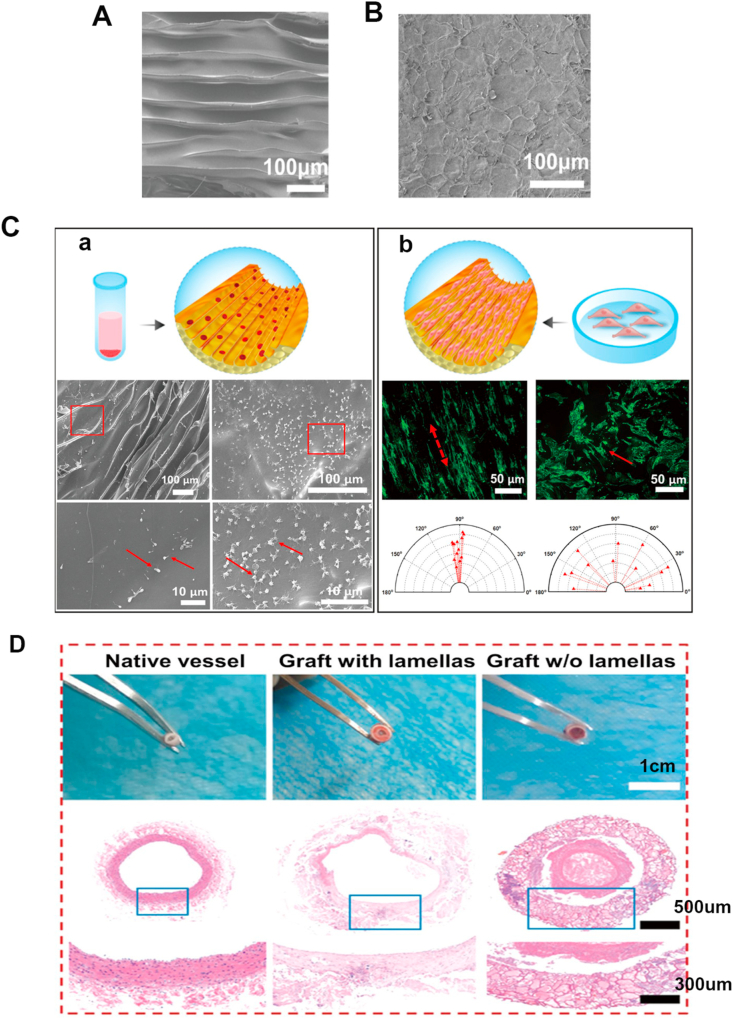
The influence of surface topography on cell morphology and biological behavior. (A–B): Scanning electron microscopy (SEM) for inner lamellar structure of vascular graft; A: Inner lamellar structure fabricated by freeze-cast, with the lamellar 10 μm high, 200 nm thick, and the interval between lamellas was 20 μm; B: Inner non-lamellar structure fabricated by direct freeze-drying. (C): Cell behavior on graft surface; (a): Platelets adhesion. SEM figures showed that less platelets adhered on lamellar structure, and they were not activated; b: ECs elongation. ECs displayed elongated adherence along aligned surface of vascular graft and enhanced proliferation. (D): Optical figures and HE staining 3 months after implantation. Reproduced from Ref. [160], ACS NANO, ACS Publication @ 2019.

Surface patterning has potential in promoting spontaneous *in situ* endothelialization [146]. Photolithography, electron beam lithography, and soft lithography can be utilized to develop micro-/nano-scale tube/groove/pillar patterns in different sizes [151,155,156]. The patterns can enhance EC elongation and endothelialization, as well as inhibit platelet adhesion and maintain long-term patency rate [144,[157], [158], [159]]. Wang et al. [160] constructed a biomimetic vascular graft modified with nano-topographic lamellar structure utilizing freeze-cast technique, and the lamella was 10 μm high, 200 nm thick (Fig. 4A). The results indicated that the nano-lamellar framework could prevent platelet activation and enhance EC orientation (Fig. 4C). The number of platelet adhesion on lamellar surface was about 3 times fewer than that on non-lamellar surface. The HE staining images indicated that lamellar structure could promote *in situ* endothelialization, while distinct thrombus formed in non-lamellar structure group (Fig. 4D). Zorlutuna et al. [161] constructed a vascular graft with channel nanopatterns at the periodicity of 650 nm. The nanopatterns were on both sides of surface to induce orientation and proliferation of ECs and SMCs simultaneously. Different surface nanopatterns are effective strategies to tune cell orientation and vascular remodeling in a pattern-dependent way.

The arrangements of adhesive peptides also influence the migration, morphology and proliferation of ECs [162]. Wang et al. [163] explored the effects of RGD nano-spacing in different nanoscale size (37–124 nm) on MSC performance, and the results indicated that RGD nano-spacing might have a role in modulating differentiation of MSCs. Saux et al. [164] found that micro-scale pyramids could impede cell migration, while RGD spacing with density of 6✕10^8^ mm^2^ could enhance cell spreading and adhesion. Karimi et al. [165] compared random and nano-clustered RGD spacing on vascular graft surface, and demonstrated that the nano-island pattern on surface could promote the migration and adhesion of ECs for enhanced *in situ* endothelialization. RGD is specific for EC capture, and designing RGD pattern on surface can further promote cell migration and proliferation.

### 3.2 Promoted cell proliferation and activation

**Fig. 5:**
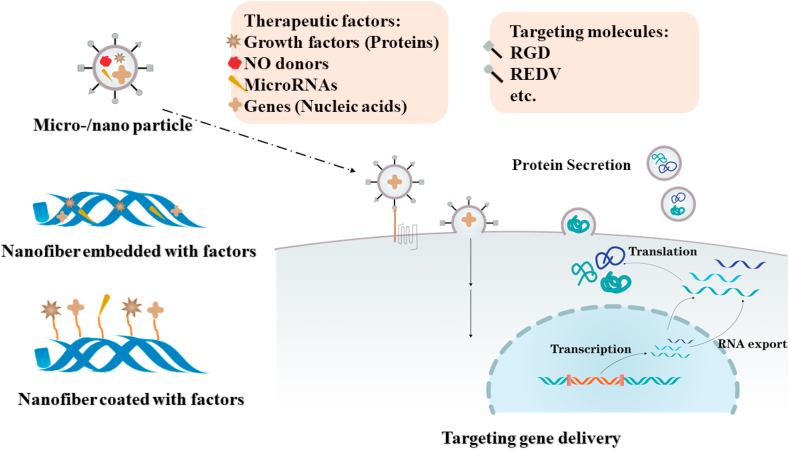
Bioactive molecules and therapeutic genes for enhanced *in situ* endothelialization. Strategies including micro/nano particle loading, nanofibers embedment or graft surface coating can be utilized to deliver therapeutic factors for promoted cell proliferation and activation. Furthermore, targeting molecules are used for more efficient gene delivery to targeted cells.

To promote the proliferation and activation of ECs and EPCs after cell adhesion, bioactive molecules and therapeutic genes are promising approaches (Fig. 5).

#### 3.2.1 Microenvironment regulation for enhanced cell performance

Multiple molecules possess capability to motivate the proliferation, differentiation and activation of ECs and EPCs, including growth factors, gas and microRNAs (Fig. 5). Vascular endothelial growth factor (VEGF) is vital in vascularization, and plays a key role in regulating EC behavior [38,166,167]. Sustained VEGF releasing in vascular graft can promote endothelialization via facilitating differentiation of EPCs and enhancing the proliferation and activation of ECs [168,169]. VEGF can be loaded on vascular grafts through multiple strategies, like NPs [170], coaxial electrospinning [171], direct blending electrospinning [172], and emulsion electrospinning [173], etc. Remarkably, VEGF can also serve as chemotaxis for EPC homing via target of VEGFR1 and VEGFR2 receptors on cell surface [174,175]. Fibroblast growth factor-2 (FGF-2), with potential in directing stem cell differentiation, is also applied in vascular graft [176,177]. Rajangam et al. [178] combined heparin and PAs, and constructed a self-assembly nanofibrous gel to capture VEGFs and FGF-2 for enhanced angiogenesis. Furthermore, platelet-derived growth factor (PDGF) can promote the migration and proliferation of SMCs [179]. Han et al. [180] constructed a double-layer electrospun nanofibrous scaffold, with inner layer loaded with VEGF for EC proliferation, and outer layer with PDGF for SMC proliferation, thus inducing an intact vascular blood vessel formation. Growth factors can directly play a role in vascular construction, but for protein delivery, protein bioactivity and concentration maintenance *in vivo* are still concerned. To enhance delivery efficiency and maintain protein activity and concentration *in vivo*, multiple nanoscale and microscale carriers are utilized, which are summarized in Table 2.

**Table 2:** Nanoscale and microscale carriers for growth factor delivery.

Carriers		Cargos	Sizes	Dosages	Results	Ref.
Nanoparticle	Chitosan/heparin NP	VEGF	67–132 nm	43, 113, or 237 ng/mL	Enhancing regeneration of decellularized tissue-engineered scaffolds	[170]
Microparticle	Alginate microbeads	FGF-1	140 μm	600 ng	Rapid and persistent vascular response	[181]
	Gelatin microspheres	FGF-2	40 μm	30 mg	Improving mechanical properties and releasing FGF-2	[182]
	Gelatin microparticles	VEGF	75–125 μm	0–100 ng/mL	Prolonging VEGF activity and increasing endothelialization	[183]
Nanofibers	Electrospun PELCL/gelatin and PLGA/gelatin nanofibers	VEGF, PDGF	VEGF 0.59 ng/mg; PDGF 0.53 ng/mg	PELCLC 568 nm; PLGA 940 nm	VEGF for EC proliferation, and PDGF for SMC proliferation	[180]
	Self-assembly nanofibrous gel	VEGF, FGF-2	25 ng	/	Promoting the tube formation of ECs	[178]

Note: Poly (ethylene glycol)-b-poly (l-lactide-co-ε-caprolactone): PELCL; poly (l-lactide-co-glycolide): PLGA.

VEGF is efficient in promoting proliferation and activation of ECs, but the immunogenicity and high expense make the application of VEGF in clinic difficult. MicroRNAs (miRNAs), the non-coding RNAs, have been reported to play a role in regulating vascularization by bonding with promotor region of target genes in recent studies [184,185]. It has been showed that miRNA-126 can regulate the vascular development via modulating the responding of ECs to VEGF, and inhibiting Spred-1 expression, which restrains angiogenic signal pathways [186,187]. Electrospun nanofibers are viable and convenient to load biomolecules, and have been widely utilized to carry miRNAs. Zhou et al. [173] utilized REDV modified PEG-trimethyl chitosan to load miRNA-126, and delivered the miRNA to the targeted ECs. The miRNA complex was incorporated into electrospun polymers utilizing emulsion electrospinning to construct vascular scaffold, and miRNA was released sustainedly for enhanced EC performance. Cui et al. [188] loaded miRNA-126 in inner electrospun fibers, and miRNA-145 in outer fibers, respectively to regulate biological behavior of ECs and SMCs. Moreover, some other miRNAs are also found and explored their effects on vascularization. MiRNA-22 could prevent the apoptosis of SMCs via targeting p38-MAPK pathway during vascular remodeling [189]. Suppression of miRNA-21 restrains EC growing through PTEN dependent-PI3K pathway [190]. Nevertheless, effects of these miRNAs are still at primary research stage, and has not been widely recognized.

Moreover, NO can also stimulate proliferation and activation of ECs, as well as homing of EPCs [191,192], which makes NO donors attractive for vascularization. The biological functions and multiple NO donors applied in vascular grafts will be introduced in the following contents.

#### 3.2.2 Therapeutic gene delivery for enhanced cell performance

Delivery of proteins like VEGF and FGF can directly bind to receptors on target cell surface, and modulate the expression of angiogenesis related genes via signaling pathways, but have risk of protein degradation, inactivation and gradual consumption. Gene therapy is a favorable approach for promoting endothelialization through transfecting ECs, since genes can be transfected into nucleus and translated for protein releasing, potential in maintaining a relative high protein concentration *in vivo* (Fig. 5) [193]. VEGF, FGF and ZNF580 genes are promising genes for therapeutic gene delivery in vascular graft applications.

To guarantee the transfection efficiency, gene carriers are vital for delivery. Nanoparticles (NPs) can act as carriers, preventing DNA from degradation by enzymes, targeting to the specific cells, entering cell membrane, translocating to the nucleus, and finally integrating to host genome. Various biomaterials have been widely utilized for gene delivery, including lipid NPs [194,195], polymeric NPs like cationic ester polymers [196], co-polymers [[197], [198], [199]], poly (ethylenimine) (PEI) [200], PEI based co-polymers [201,202], inorganic NPs like calcium phosphate [203], and peptide based NPs [204]. Furthermore, electrospinning technology is also attractive in gene loading. Plasmid-DNA (pDNA) can be directly mixed in solvent for electrospinning [203,205,206], or modify nanofibers with gene loaded microparticles [207].

The cell adhesive peptide like RGD [208,209], REDV [210] can be utilized for target gene delivery to facilitate peptide modified pDNA complex bind to integrins on EC surface. Wang et al. [210] constructed a REDV modified pZNF580 NP complexes utilizing self-assembling strategy. The REDV mediated NPs could prevent the pZNF580 from DNase degradation, display better hemocompatibility and enhance delivery efficiency for enhanced *in situ* endothelialization. Kibria et al. [211] utilized RGD and PEG as dual-ligand modification to promote target gene delivery efficiency.

Delivered genes can integrate into target cell genome, and stably expressing transfected proteins in a long period time, compared with direct protein delivery. But the transfection efficiency is a potential risk, and the transferred genes cannot function *in vivo* immediately like proteins.

## 4 Preventing vascular incidents for long-term lumen patency

Thrombogenesis, IH and calcification can reduce lumen diameter, and are major risks in maintaining long-term lumen patency after vascular implantation. Preventing thrombus formation, IH and calcification is crucial for survival of vascular graft. Hydrophilic surface and heparin coating are effective in anti-thrombogenesis, and some drugs can inhibit IH. Moreover, NO can play a role in inhibiting coagulation and IH.

### 4.1 Anti-thrombogenesis for enhanced long-term lumen patency

It is easy to provoke thrombogenesis if lacking ECs on graft surfaces, but during early healing process, there have not been adequate ECs lining on surface to release molecules for thrombus prevention. Thus, at the beginning of implantation, vascular grafts may direct contact with blood cells in vasculature. The vascular grafts are transplanted as foreign matters, and easy lead to the absorption of plasma proteins and blood cells, and then activate the coagulation cascades [212]. The graft surface with minimized protein adsorption, drugs or gases for anti-coagulation are effective strategies for thrombogenesis prevention.

To inhibit thrombogenesis, minimizing the absorption of plasma proteins on graft surfaces is crucial. Thus, a biocompatible surface with minimized protein adsorption is required. The hydrophilic surface can effectively prevent the protein adsorption. Some biocompatible and hydrophilic biomaterials have been utilized for the surface modification of vascular grafts, for example, PEG [213], zwitterionic polymers [214,215]. PEG and zwitterionic polymers or groups can be directly coated [216], blended [217,218] or covalently grafted [213,219] on the scaffold surfaces, and the hydrophilic surfaces can effectively inhibit the protein adsorption and platelet adherence [214,220].

Furthermore, heparin, known as an anti-coagulative drug, can be coated or immobilized on vascular graft surface, which plays a role in anti-thrombogenesis [[221], [222], [223]]. Heparin can interact with anti-thrombase AT III, to restrain the function of thrombase and coagulation factor Xa. Heparin, with carboxyl groups, can also be blended with gelatin to provide a biocompatible and anti-coagulative surface [88,224]. Liu et al. [225] constructed heparin/poly-l-lysine mixed NPs, and immobilized these NPs on a dopamine modified surface. They found that heparin modified surface could enhance biocompatibility, inhibit fibrin induced platelet adherence, and prolong thrombin time (TT) to 23.7–27.9s. Moreover, LBL technology can be utilized to graft heparin on the graft surfaces [226]. Easton et al. [227] constructed a LBL coating, utilizing ploy (acrylic acid) and polyethyleneimine to immobilized heparin on an electrospun nanofibrous scaffold, and the results indicated that the hemocompatibility of scaffold was enhanced.

Some other anti-coagulation drugs can also be utilized, for example, clopidogrel, warfarin, t-PA [228]. The t-PA is the enzyme converting plasminogen to plasmin, inducing the fibrinolytic process [229]. Liu et al. [228] immobilized t-PA on electrospun PVA nanofibrous mats, aiming to mimic natural fibrinolysis function and prevent thrombus formation. Furthermore, Gastrodin, which is applied in cardiovascular diseases, can lower blood viscosity and regulate inflammation reactions. Zheng et al. [25] fabricated Gastrodin-loaded PU scaffolds, and found that the Gastrodin modified grafts showed greater potential in preventing thrombogenesis and inflammations.

Apart from surface modification and drugs, one novel nanocomposite has been indicated to possess the ability to prevent thrombogenesis and display ideal mechanical characteristics [[230], [231], [232]]. To inhibit thrombogenesis of PU vascular graft scaffolds and improve bistability of PU *in vivo*, Kannan et al. [233] attached polyhedral oligomeric silsesquioxane (POSS) nanoparticles into poly (carbonate urethane) (PCU) scaffold with covalent bonding and developed a new POSS-PCU nanocomposite biomaterial. This POSS-PCU nanocomposite can be developed into small-diameter vascular grafts with enhanced mechanical properties and hemocompatibility, free from adverse events like IH and calcification.

For hydrophilic surface, it can reduce fibrin adherence and coagulation cascades, but can also decrease EC adhesion. For heparin modification, the bioactivity and coating density are concerned. Hence, more studies are required to obtain ideal graft surface with reduced platelet and fibrin adherence and enhanced EC adhesion.

### 4.2 Anti-IH for enhanced long-term lumen patency

IH has high incidence in causing long-term vascular occlusion several months after implantation, which is caused by uncontrollable SMC pathological proliferation [234].ECs, serving as the paramount defender in vascularization, can release molecules for IH inhibition. Apart from promoting EC adhesion and activation, some drugs like E2F and MK2i also have potential in preventing IH by controlling SMC proliferation in a more direct way [235]. In clinic, E2F transcription factor was utilized to keep a 30 min lumen patency for the vascular tissue *in vitro*. But the clinical trial results indicated that E2F treated grafts *in vitro* failed to preventing IH via inhibiting proliferation of SMCs after implantation [236,237]. The p38 MAPK signaling pathway can play a role in activating proliferation of SMCs, and then induce the downstream inflammatory and fibrotic cascades in IH [[238], [239], [240]]. To prevent IH, Evans et al. [241] utilized MAKP inhibitory peptide (MK2i) and constructed MK2i NPs for graft modification to prevent uncontrollable proliferation of SMCs.

### 4.3 Role of NO in enhancing long-term lumen patency

**Fig. 6:**
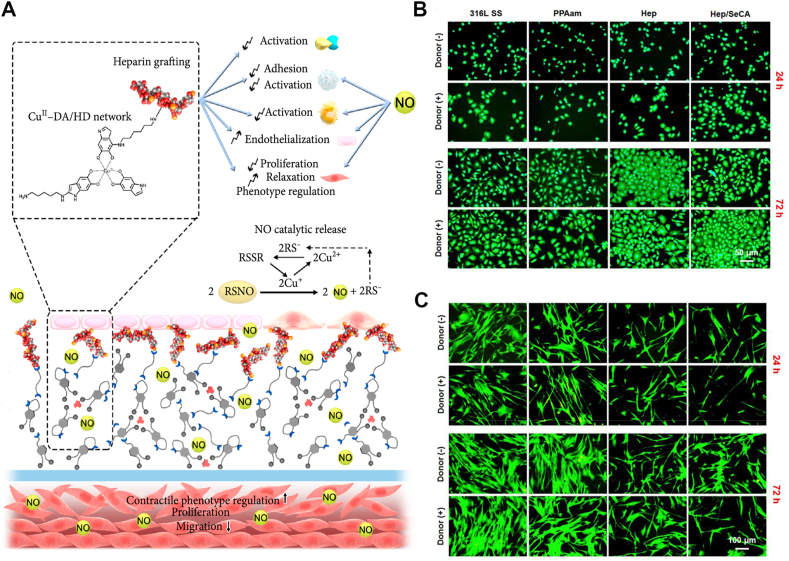
NO plays a crucial role in modulating endothelialization, thrombogenesis and IH. (A): The biological performance of NO. NO can be liberated by catalyzing NO donors, and play a role in vascularization, including inhibiting activation of thrombin, platelets, immune cells and proliferation of SMCs, as well as promoting proliferation and activation of ECs, relaxation and phenotype regulation of SMCs. (B–C): Fluorescence staining of ECs and SMCs 24h and 72h after *in vitro* culture. NO can promote EC proliferation (B) and inhibit SMC growth (C). (A) reproduced from Ref. [242], Research, CAST@ 2020. (B–C) reproduced from Ref. [249], Biomaterials, Elsevier @ 2019.

NO can play an important protective role in vasculature, and also play an effective role in influencing relevant physiological functions (Fig. 6A) [191]. In physiological environment, NO is secreted by endothelial cells, which is synthesized through catalytic reactions in the presence of nitric oxide synthase (NOS) [242]. NO can inhibit the aggregation and activation of platelets, to avoid thrombogenesis [243], restrain proliferation of SMCs, to avoid IH [242,244], as well as prohibit the recruitment and activation of inflammatory cells, to avoid inflammation (Fig. 6A) [245]. Furthermore, NO plays a critical role in promoting the growth of endothelial cells (Fig. 6A) [246]. Insufficient ECs lead to deficient NO release, which may then trigger pathological process [247]. Hence, abundant NO release is required to enhance ECs growth, inhibit SMCs proliferation, and provide an appropriate environment for endothelialization of vascular graft [248].

N-diazeniumdiolates (NONOates) and S-nitrosothiol (RSNOs) can serve as the potential NO donors to be applied in biomaterials. One mole of NONOate [^−^N(O) = NO^−^] can be catalyzed to release 2 mol NO via the hydrolysis reactions in the physiological environment (temperature at 37 °C, pH at 7.4) [250]. In RSNOs, thiols (-SH) can react with nitrous acid (HNO_2_) to release NO, generally under the catalysis of copper ion (Cu^2+^) [251,252]. Selenocystamine (SeCA), having glutathione peroxidase (GPX)-like functions, is also a potential catalysts for NO synthesis by decomposition of RSNOs [249,253,254].

Multiple strategies have been utilized in vascular devices to catalyze NO generation, like selenocystamine (SeCA) [249,254], metal-phenolic surface [253], and copper ion (Cu^2+^) [242,255]. Qiu et al. [249] utilized SeCA coating to catalyze NO generation, and the results indicated that NO can promote EC proliferation and inhibit SMC growth (Fig. 6B and C). The amount of ECs on SeCA/heparin surface was about 1.5–2.0 folds more than that on naked surface, and EC migration distance and density was enhanced by 26% and 23%, respectively on SeCA/heparin compared with the naked one [249]. Nanoparticles (NPs) can serve as effective delivery vehicles for NO release. The better surface to volume rate can offer more chance for optimized quantity of NO donors. Targeted NPs can deliver NO via altering surface chemical characteristic to regulate the localized areas [256]. Various NPs have been studied to serve as carriers for NO donors, including silica nanoparticles (SiNPs) [[257], [258], [259]], liposome nanoparticles [260,261], and metallic nanoparticles [255].

SiNPs are easily compounded in the nanoscale, and contain functional groups like amine groups (-NH_2_) on the surface [262]. The amine-functionalized SiNPs were synthesized, with the size ranging from 20 to 500 nm, to serve as NO carriers via converting the –NH_2_ groups into NONOates donors under high pressure of NO [262]. To promote the storage and releasing characteristics of NO, the synthesis of SiNPs following the preparation of NONOates modified aminosilanes was proposed by the same team [263]. Fumed silica (FS) particles can also serve as NO carrier, and can be embedded into polymers to control NO releasing [258]. Zhang et al. [259] synthesized FS particles (200–300 nm), with NONOates formed on the surface, and embedded within PU films as anti-thrombotic coating.

Liposomes are competent carriers, owing to their efficient cell encapsulation ability [264]. The influence of hydrophobic liposomes and surface micelles on the NO releasing were studied. Dinh et al. [261] found that anionic 1,2-dipalmitoyl-sn-glycero-3-[phospho-(1-glycerol)] sodium salt (DPPG) liposomes possessed greater NO releasing catalysis efficiency than sodium dodecylsulfate (SDS) micelles. Dinh et al. [265] further explored the influence of unilamellar (anionic and cationic) phospholipid vesicles on dissociating NO from NONOates, and found that anionic liposome NPs showed an enhanced NO releasing. Thermosensitive liposome NPs [266] and photo-sensitive NO donors [267] were also explored.

Silica and liposome NPs described above can effectively load and liberate NO, but cannot deliver the gas to targeted tissues. Some metallic NPs are potential strategies to serve as loading vehicles for NO delivery to targeted tissues, including gold (Au) NPs [[268], [269], [270]], platinum (Pt) NP [271], silver (Ag) NPs [[272], [273], [274]]. Furthermore, metal organic frameworks (MOFs), composed of metal ions as nodes and organic ligands as linkers, have been utilized to embed and release NO to promote re-endothelialization for vascular grafts [275]. Fan et al. [255] constructed nanoscale copper-based MOFs (Cu-MOFs), and proved that Cu-MOFs performed as heterogeneous catalysts for NO regeneration synthesized by endogenous RSNOs. Simultaneous delivery of NO and Cu^2+^ could restrain restenosis and enhance endothelialization synergistically [275].

Moreover, NO gas can be combined with growth factor VEGF, and spontaneously released to promote endothelialization and inhibit thrombogenesis and IH [276]. Although multiple studies have proved the influence of NO in *in vitro* and in *vivo* experiments, no clinical trials in therapeutic effects of NO donors have been conducted. Furthermore, the therapeutic effects on diabetic wounds still need more concerns [193].

## 5 Immunomodulation in vascular graft development

Inflammatory responses, induced by graft implantation, are crucial in modulating graft development. Molecules released from immune cells influence the biological behavior of ECs and SMCs, and thus modulating *in situ* endothelialization and lumen patency of vascular graft.

### 5.1 Immunomodulation and *in situ* endothelialization

Macrophages, as the key cells in innate immunity, can release multiple molecules modulating *in situ* endothelialization process [277,278]. It has been reported that TNF-α secreted by macrophages plays a role in regulating migration and differentiation of EPCs and graft development. TNF-α can induce the differentiation of EPCs through activating TNF-α receptor 1 and NF-κB signaling pathway [279]. Moreover, TNF-β1 can promote platelet mediated-EPC homing via integrin β3 on cell surface [280].

The polarization of macrophages can be controlled to regulate the inflammatory microenvironment. Classical macrophages (M1) may secret inflammation cytokines, while alternatively induced macrophages (M2) tend to activate anti-inflammation molecules and enhance tissue repairment [281]. The acute inflammation responses can be modulated by lipid mediators which possess potential in anti-inflammation, like Resolvins [282,283]. Shi et al. [284] incorporated Aspirin-Triggered resolvin D1 (AT-RvD1) into electrospun PCL vascular graft, and demonstrated that the incorporation of AT-RvD1 enhanced vessel tube formation *in vitro* through M2 macrophage polarization.

Inflammatory cells paly a complex role in regulating angiogenesis. VEGF-A is an important modulator in vascularization, and recently one subset type of neutrophils (which was shown as CD49d^+^VEGFR1^high^CXCR4^high^) were identified [285]. Massena et al. [285] demonstrated that homing this subset neutrophils to hypoxia neovascularization site could promote angiogenesis. The interaction between angiogenic cells and inflammatory cells is still not completely clarified, which requires more further explorations.

### 5.2 Immunomodulation and long-term lumen patency

Inflammatory cells also play a role in modulating the long-term lumen patency after graft implantation, like IH and intima calcification. Molecules secreted by macrophages and platelets, like TGF-β, cause enhanced migration and proliferation of SMC, and the unregulated SMC proliferation and the inrush of macrophages are dominating factor leading to IH [286]. SMCs from anastomosed sites migrate towards intima, and SMCs transform from the quiescent phenotype into the dedifferentiated, proliferative type, thus resulting in IH [[287], [288], [289]].

Macrophages are the predominant inflammatory cells targeting SMCs, and lead to the pathological proliferation of SMCs [290,291]. Thus, reducing IH via regulating macrophage behavior has attracted great attention. To attenuate chronic inflammation induced IH, Ding et al. [292] constructed resveratrol (RSV) modified carbon nanotube (CNT) coating for tissue engineered blood vessels. RSV has been reported to inhibit inflammation process via inducing M2 macrophage polarization [293]. The RSV modified CNT can be utilized for macrophage intake, and released intracellularly, which effectively promoted transformation of M1 into M2 and prevented IH [292]. Yang et al. [294] modified a decellularized vascular graft with the rapamycin (RM)-blended electrospun PCL coating, and the results indicated that the RM-loaded graft could enhance M2 polarization, inhibit IH, and promote endothelialization.

Inflammation is also the leading factor contributing to atherosclerotic calciﬁcation [295]. Wei et al. [296] extracted small extracellular vesicles (sEVs) containing VEGF, miRNA126, and miRNA145 from MSC, and loaded sEVs on a heparinized electrospun PCL scaffold. Wei et al. [296] found that sEVs loaded graft exhibited immunomodulatory function, and induced the transition of M1 into M2, which effectively reduced graft calcification and enhance lumen patency for hyperlipidemia patients.

**Fig. 7:**
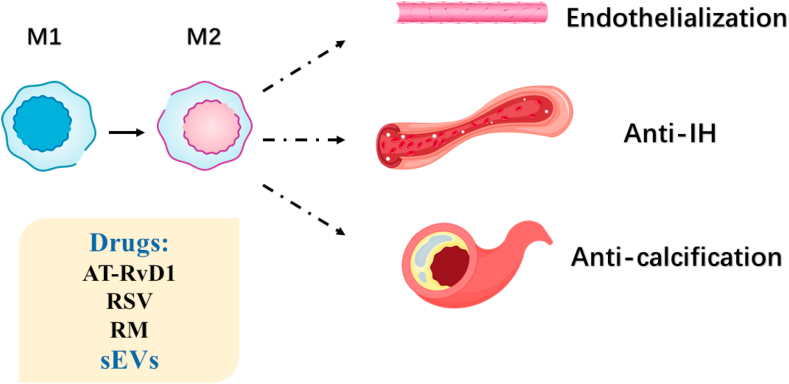
Macrophage performance in vascularization. Drugs or sEVs are utilized to promote the transition of M1 into M2 and regulate inflammation reactions for endothelialization enhancement, anti-IH and anti-calcification.

Macrophages, the key cell in innate immune system, can polarize from M1 type to M2 type, which is induced by biomaterials, drugs or sEVs, to regulate behavior of ECs and SMCs, thus promote endothelialization and inhibit IH and calcification (Fig. 7). NK cells also play a role in vascular remodeling. BALB/c mice lack in NK functions display distinctly less IH occurrence [297], but approaches to regulate NK cell functions using bioactive scaffolds have not been available. Generally, the role of complements and cytokines in innate immune system are understood. However, the performance of adaptive immune system, including T cells, B cells and mast cells, is not that clear. For better modulation of immune functions, more studies on regulation of adaptive immune cell behavior are required.

## 6 Conclusions and further perspectives

The challenges for *in situ* endothelailization and long-term patency of small-diameter vascular grafts still exist. ECs form the inner endothelium layer, which play a crucial role in maintaining vascular hemostasis and lumen patency, but homing and capture of EPCs and ECs for conventional graft is poor. Naked graft surface without lined EC layer is easy to be deposited with blood cells, fibronectin, and platelets, thus inducing coagulation cascades and thrombogenesis. Furthermore, uncontrollable proliferation of SMCs migrating to intimal layer may result in IH, and inflammatory cells also play a role in regulating biological behavior of ECs, EPCs, and SMCs.

**Fig. 8:**
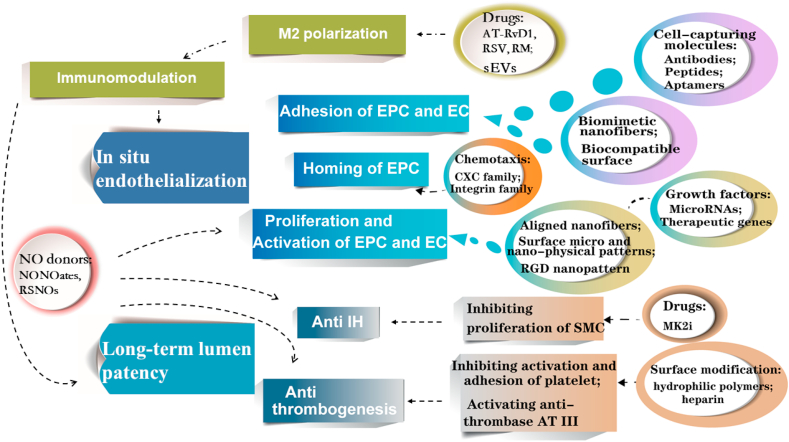
Multiple strategies for enhanced *in situ* endothelialization and long-term lumen patency.

Multiple strategies can be adopted for enhanced *in situ* endothelialization and long-term patency of vascular grafts (Fig. 8). Strategies for *in situ* endothelialization promotion, thrombogenesis and IH prevention, and immunomodulation in vascular graft remodeling are summarized as following.

(1) Strategies for enhanced *in situ* endothelialization:
1) **Homing of EPCs**: Chemokines aimed at ligands like CXC family and integrin family on EPC surface can be utilized for EPC homing.
2) **Migration and adhesion of EPCs and ECs**: Nanofibrous structure, biocompatible surface for more binding sites like gelatin and cell-capturing molecules on graft surface including antibodies, specific peptides and aptamers can be applied for better cell adhesion.
3) **Proliferation and activation of EPCs and ECs**: Topography of scaffold can regulate cell orientation, like aligned nanofibers, surface micro-/nano-patterns, and RGD patterns on surface. Growth factors, microRNAs and therapeutic genes can modulate cell bioactivity.
(2) Strategies for long-term patency:
1) **Preventing thrombogenesis**: Surface modification with heparin or hydrophilic polymers can inhibit activation and adhesion of platelets, as well as activate AT III, thus reduce thrombogenesis.
2) **Preventing IH**: Some drugs releasing like MK2i can inhibit IH via inhibiting proliferation of SMCs.
3) **The role of NO**: NO also plays a crucial role in vascular graft remodeling. NO is potential in inhibiting activation of thrombin, platelets, immune cells and proliferation of SMCs, as well as promoting proliferation and activation of ECs.
(3) Strategies for modulating immunomodulation:

**Immunomodulation**: Some drugs like AT-RvD1, RSV and RM promote M2 polarization, and exert influences on behavior of ECs and SMCs. sEVs can be utilized for M2 polarization and prevent calcification.

Multiple strategies have been explored to promote *in situ* endothelialization, inhibit thrombogenesis and IH, but the approaches are limited to experimental researches. More researches concerning toxicity, mechanical properties, degradation rate and delivery efficiency should be considered and conducted for further application in clinics.

## Author contributions

Y.Z., C.Z., and M.C. collected references, summarized perspectives and wrote the manuscript. J.H. and Q.L. collected references. G.Y. gave the suggestion and help on the revision of this review. K.L. and Y.H. conceived the concept of this review. All authors discussed and commented on the manuscript.

## Declaration of competing interest

The authors declare that they have no known competing financial interests or personal relationships that could have appeared to influence the work reported in this paper.
